# Minnelide effectively eliminates CD133^+^ side population in pancreatic cancer

**DOI:** 10.1186/s12943-015-0470-6

**Published:** 2015-11-23

**Authors:** Alice Nomura, Olivia McGinn, Vikas Dudeja, Veena Sangwan, Ashok K. Saluja, Sulagna Banerjee

**Affiliations:** Department of Surgery, Division of Basic and Translational Research, University of Minnesota, Minneapolis, MN 55455 USA; Masonic Cancer Center, University of Minnesota, Minneapolis, MN USA

## Abstract

**Background:**

Pancreatic Ductal Adenocarcinoma (PDAC) is a devastating disease hallmarked by limited patient survival. Resistance to chemotherapy, a major cause of treatment failure in PDAC patients, is often attributed to Cancer Stem Cells (CSCs). Pancreatic CSCs are a small subset of quiescent cells within a tumor represented by surface markers like CD133. These cells are responsible not only for tumor recurrence, but also poor prognosis based on their “stem-like” characteristics. At present, conventional therapy is directed towards rapidly dividing PDAC cells and thus fails to target the CSC population.

**Methods:**

MIA PaCa-2, S2-013 and AsPC-1 were treated with 12.5 nM triptolide (12 T cells) for 7 days. The surviving cells were recovered briefly in drug-free growth media and then transferred to Cancer Stem cell Media (CSM). As a control, untreated cells were also transferred to CSM media (CSM). The 12 T and CSM cells were tested for stemness properties using RNA and protein markers. Low numbers of CSM and 12 T cells were implanted subcutaneously in athymic nude mice to study their tumorigenic potential. 12 T and CSM cells were sorted for CD133 expression and assayed for their colony forming ability and sphere forming ability. Invasiveness of 12 T cells, CSM and MIA PaCa-2 were compared using Boyden chamber assays.

**Results:**

Treated 12 T cells displayed increased expression of the surface marker CD133 and the drug transporter ABCG2 compared to untreated cells (CSM cells). Both 12 T and CSM cells formed subcutaneous tumors in mice confirming their tumor-initiating properties. When tested for invasion, 12 T cells had increased invasiveness compared to CSM cells. CD133^+^ cells in both CSM and 12 T showed greater colony and sphere forming ability compared to CD133^−^ cells from each group. Consistent with these data, when injected subcutaneously in mice, CD133^−^ cells from CSM or 12 T did not form any tumors whereas CD133^+^ cells from both groups showed tumor formation at a very low cell number. Despite pre-exposure to triptolide in 12 T CD133^+^ cells, treatment of tumors formed by these cells with Minnelide, a triptolide pro-drug, showed significant tumor regression.

**Conclusion:**

Our results indicated that triptolide enhanced and enriched the “stemness” in the PDAC cell lines at a low dose of 12.5 nM, but also resulted in the regression of tumors derived from these cells.

**Electronic supplementary material:**

The online version of this article (doi:10.1186/s12943-015-0470-6) contains supplementary material, which is available to authorized users.

## Background

Pancreatic cancer is one of the most aggressive malignancies with an extremely poor survival rate [1]. Even for patients who undergo potentially curative resection, the 5-year survival is less than 5 % due to local recurrence and metastasis [2, 3]. Many different chemotherapeutic agents, including the current standard of care, Gemcitabine, have failed to demonstrate any significant survival advantage in patients with pancreatic adenocarcinoma. Emerging evidence has shown that cancer stem cells (CSCs), a small subset of quiescent cells within a tumor, are responsible for tumor recurrence [4].

The significance of CSCs in hematological malignancy as well as in solid cancers is well known [5]. Pancreatic cancer stem cells (PCSC) were identified in 2007, when several groups demonstrated the presence of CD24, CD44, epithelial specific antigen (ESA) triple positive markers or CD133 positive cells had the ability to initiate tumor formation in animals at very low numbers [6, 7]. Since then, many such surface markers have been identified [8–10]. These tumor initiating cells (TICs) or CSCs are thought to be responsible for not only tumor recurrence but also chemo-resistance and metastatic spread of a tumor. Pancreatic cancer stem cells have been reported to be resistant to gemcitabine induced apoptosis [7]. Later, Shah et al [11] and Du et al [12] established gemcitabine-resistant pancreatic cancer cell lines and found that resistant cells comprised of more cells with cancer stem cell-like phenotypes compared to the parental cells.

Expression of the TIC marker CD133 in several cancers is shown to be associated with increased expression of drug transporters like ABCG2 [13, 14]. Similarly, treatment with low concentrations of a chemotherapeutic agent like gemcitabine has been reported to enrich for CSC-like properties in a number of cancers [11, 15, 16]. Chemo-resistant CSCs in a tumor can be characterized by “Side Population (SP) analysis” [17]. SP cells can rapidly efflux lipophilic fluorescent dyes to produce a characteristic profile based on fluorescence-activated flow cytometric analysis [18, 19]. Although representing only a small fraction of the whole cell population, they appear to be enriched in stem-like cells that can initiate tumors. Thus, they could provide a useful tool and a readily accessible source for cancer stem cell studies [20, 21]. SP cells identified in bone marrow obtained from patients with acute myeloid leukemia (AML) are candidate leukemic stem cells [22, 23]. They have also been identified in various human solid tumors and cancer cell lines [12, 24]. However, the reports of their presence within pancreatic tumor have been rare.

Apart from imparting chemo-resistance to tumors, cancer stem cells and SP cells are also associated with increased invasiveness [24]. CD133 expression, for example, has been correlated with increased metastasis and poor prognosis in a number of cancers like colorectal cancer and hepatocellular carcinoma [25, 26]. In pancreatic cancer, CD133^+^CXCR4^+^ population and CD44^+^Met^+^ population show increased metastatic potential when compared to the respective negative population [7, 27]. Consistent with this, genes involved in epithelial-mesenchymal-transition (EMT) are overexpressed in cancer stem cells resulting in their increased invasiveness [28, 29]. Further, recent evidence has demonstrated the surface expression of CD133 induces this invasive phenotype through the activation of NF-kB signaling [14].

Triptolide, a diterpene triepoxide, and its water-soluble prodrug, Minnelide, have been very effective in a number of preclinical cancer models, including pancreatic cancer murine models [30–32]. Recently, we demonstrated that Minnelide is effective in not only reducing the bulk tumor, but also in targeting the tumor initiating CD133^+^ population in pancreatic cancer [33]. However, the effect of triptolide on drug resistant side-population has not been tested.

In the current study, we have developed a model in which treatment with very low dose of triptolide of pancreatic cancer cell lines MIA PaCa-2, S2-013 and AsPC-1 results in a “drug-tolerant” population of cells that are enriched for most “stemness” or tumor initiating cell (TIC) markers for pancreatic cancer. These cells demonstrated increased DNA dye efflux and formed tumors at low cell concentration in athymic nude mice. Furthermore, these cells showed increased invasive properties and increased expression of EMT genes. Interestingly, when treated with Minnelide, these tumors showed complete regression, indicating that Minnelide can effectively target pancreatic cancer stem like cells.

## Results

### Long-term triptolide treatment enriches for SP cells

**Fig. 1:**
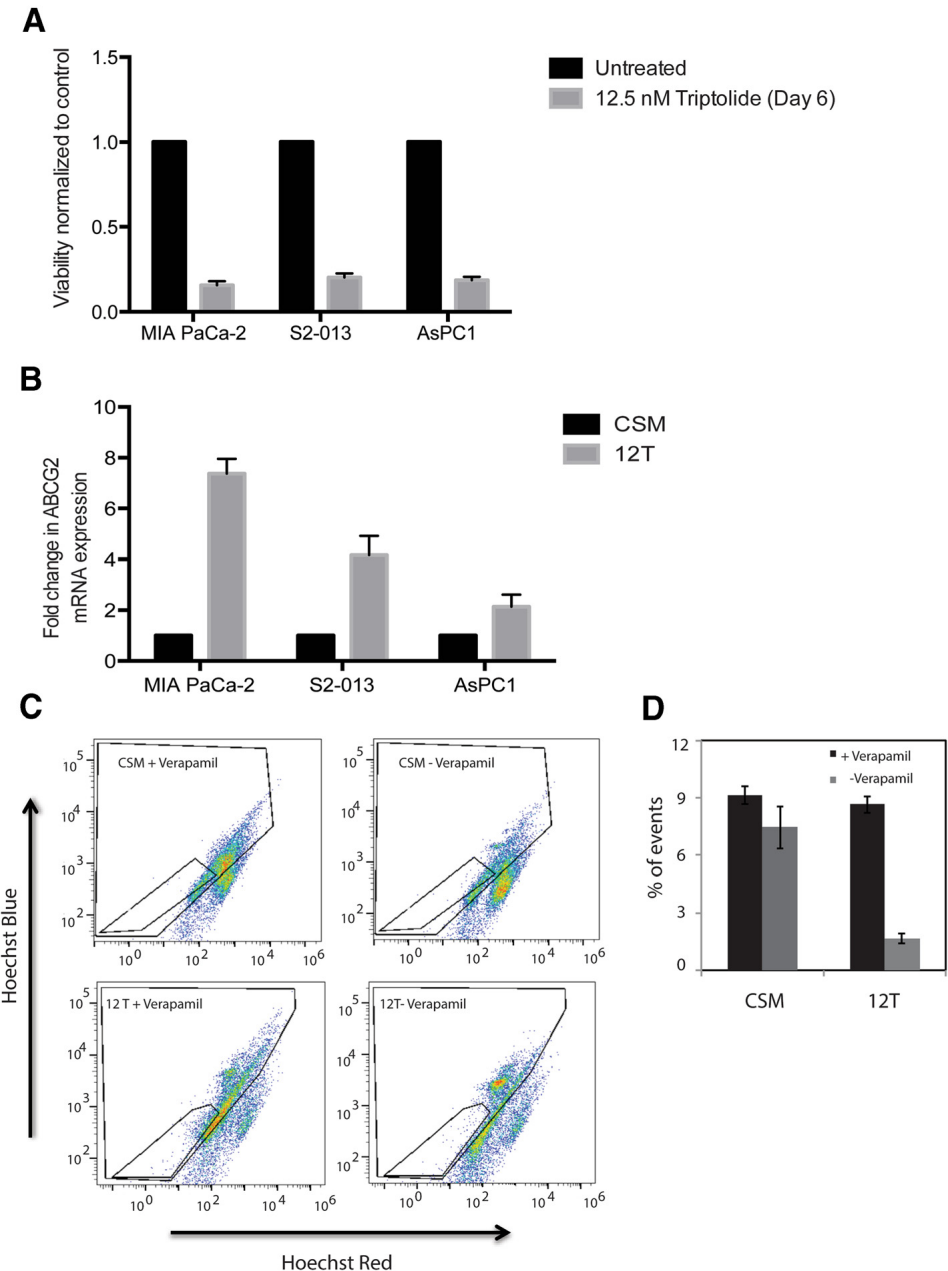
**a** Treatment of pancreatic cancer cell lines MIA PaCa-2, S2-013 and AsPC1 with 12.5 nM triptolide for 7 days resulted in 20 % viable cells. **b** These cells (12 T) had increased expression of ABCG2 gene when compared to untreated CSM cells. **c** Increased expression of ABCG2 correlated with increased DNA dye efflux in MIA PaCa-2 12 T cells. **d** Quantitation of inhibition of DNA dye efflux on treatment with Verapamil, an inhibitor of ABC transporter in 12 T and CSM cells. The error bars represent SEM (*n* = 4; * = *p* < 0.05)

To determine if exposure to a very low dose of triptolide was effective in enriching for the chemo-resistant side population in pancreatic cancer cell lines, we treated pancreatic cancer cell lines MIA PaCa-2, S2-013 and AsPC-1 with 12.5 nM triptolide for 7 days. At the end of this study, approximately 20 % of cells were viable (Fig. 1a). These cells (12 T) showed increased expression of the drug transporter gene ABCG2 (Fig. 1b) compared to the control cells (CSM). To evaluate if expression of drug transporter genes was consistent with the DNA dye efflux property of these cells, we measured the ability of 12 T cells to efflux the DNA dye Hoechst 33342. As expected, 12 T cells showed increased DNA dye efflux property compared to the CSM cells and were thus similar to the “side-population” or SP cells (Fig. 1c and d).

### Side population cells are enriched for CSC-like properties

**Fig. 2:**
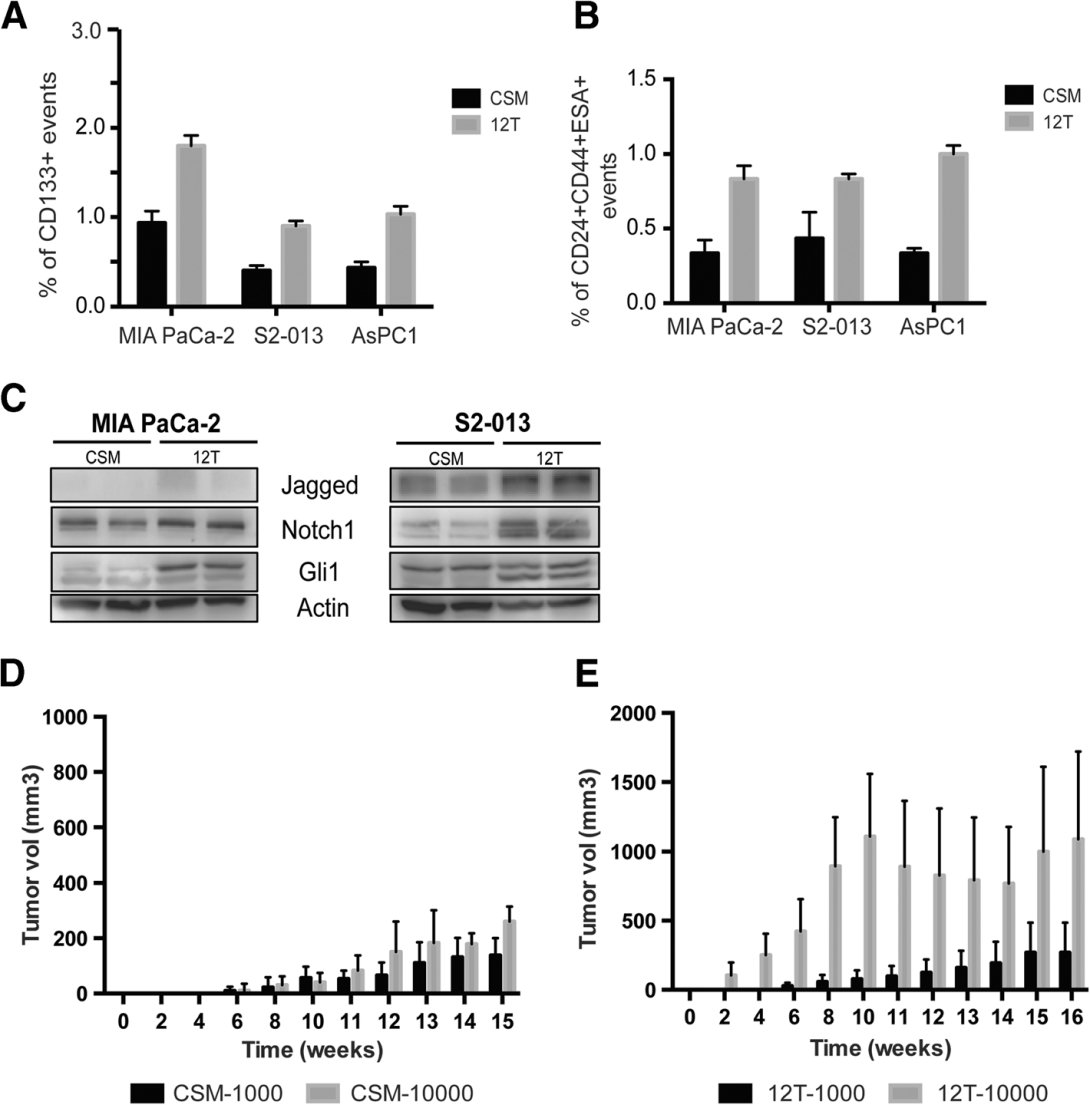
**a** 12 T cells showed higher percentage of CD133^+^ cells in all three cell lines tested: MIA PaCa-2, S2-013, AsPC1. **b** 12 T cells also showed increased CD24^+^CD44^+^ESA^+^ cells compared to CSM cells. CSM and 12 T cells showed increased expression of stemness proteins like Jagged, Notch1 and Gli1 (**c**) Tumor progression in athymic nude mice from (**d**) CSM cells and (**e**) 12 T cells. The error bars represent SEM (*n* = 4; * = *p* < 0.05)

SP cells generated as described in the previous section were assayed for previously identified pancreatic cancer stem cell markers. 12 T cells from all cells lines showed enrichment for CD133 (Fig. 2a) and CD24^+^CD44^+^ESA^+^ populations (Fig. 2b) compared to parental cell lines (MIA PaCa-2, S2-013 and AsPC-1). Additionally, 12 T cells also showed increased expression of the stemness genes Sox2, Oct4 and Nanog as well as several developmental genes known to be deregulated in cancer stem cells, such as SHH, Notch1, Gli1, and Jagged gene expression (Additional file 1: Table S1) and protein expression (Fig. 2c).

Since the definitive test of stemness is their ability to form tumors in animals, we injected 1000 and 10,000 MIA PaCa-2, CSM-MIA and 12 T-MIA cells in the flanks of athymic nude mice. Mice with MIA PaCa-2 cells did not form tumors until the end of the study (*n* = 5), whereas mice with CSM cells showed a tumor take of 11 % (1 of 9 formed tumors) in the 1000 cell group and 22 % (2 of 9 formed tumors) in the 10,000 cell group (Fig. 2d, e). Maximum tumorigenicity was seen in the 12 T group where mice receiving 1000 cells showed a 44 % tumor take (4 of 9 mice formed tumors) and mice receiving 10,000 cells showed 87 % tumor take (7 of 8 mice formed tumors) (Fig. 2d, e; summarized in Additional file 2: Table S2).

### Triptolide enriched CD133^+^ cells show increased stemness and tumor initiation properties

Since treatment with low concentration of triptolide enriched for CD133^+^ cells in pancreatic cancer cell lines, we next sorted for CD133^+^ cells and evaluated stemness properties using the classical colony forming and sphere forming assays *in vitro* and tumor forming activity in vivo.

**Fig. 3:**
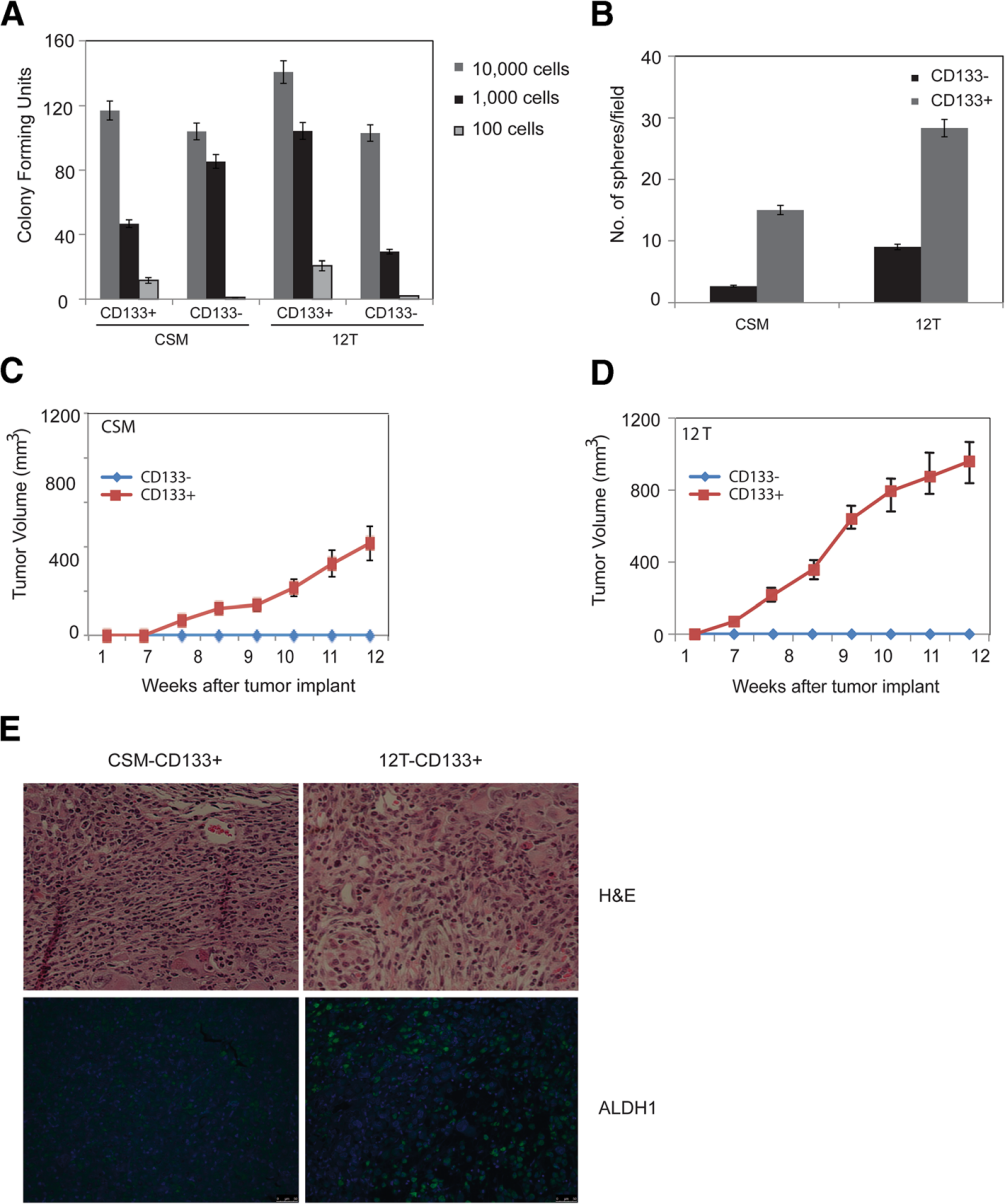
CD133^+^ cells from both CSM and 12 T cells showed **a** increased colony forming ability and **b** increased sphere forming ability when compared with CD133^−^ cells. Tumor volume over time from **c** 500 CSM CD133^+^ cells and **d** 500 12 T CD133^+^ cells formed tumors in athymic mice while the CD133- cells did not form tumors in either group. **e** H&E staining and immunofluorescent staining of ALDH1 of the CSM-CD133^+^ and 12 T-CD133^+^ derived tumors. The error bars represent SEM (*n* = 4; * = *p* < 0.05)

Sorted CD133^+^ cells from both CSM and 12 T formed colonies at high cell dilutions (100 cells) where as CD133^−^ population did not (Fig. 3a). Similarly, sphere formation was observed in CD133^+^ population in both CSM and 12 T where as CD133^−^ population from either group did not (Fig. 3b). To study if CD133^+^ population was tumorigenic, we sorted cells based on CD133 expression and implanted them subcutaneously in the flanks of athymic nude mice. Consistent with the *in vitro* surrogate experiments, CD133^+^ cells from both CSM and 12 T groups formed tumors in mice where as CD133^−^ cells did not (Fig. 3c, d). The tumorigenicity of the 12 T and the CSM cells is tabulated in Additional file 3: Table S3. The H&E staining of the tumors obtained from CSM or 12 T cells did not show any major difference in histology however increased ALDH1 staining was observed in tumors derived from 12 T cells (Fig. 3e).

### Triptolide enriched cells show increased invasiveness and increased expression of EMT genes

**Fig. 4:**
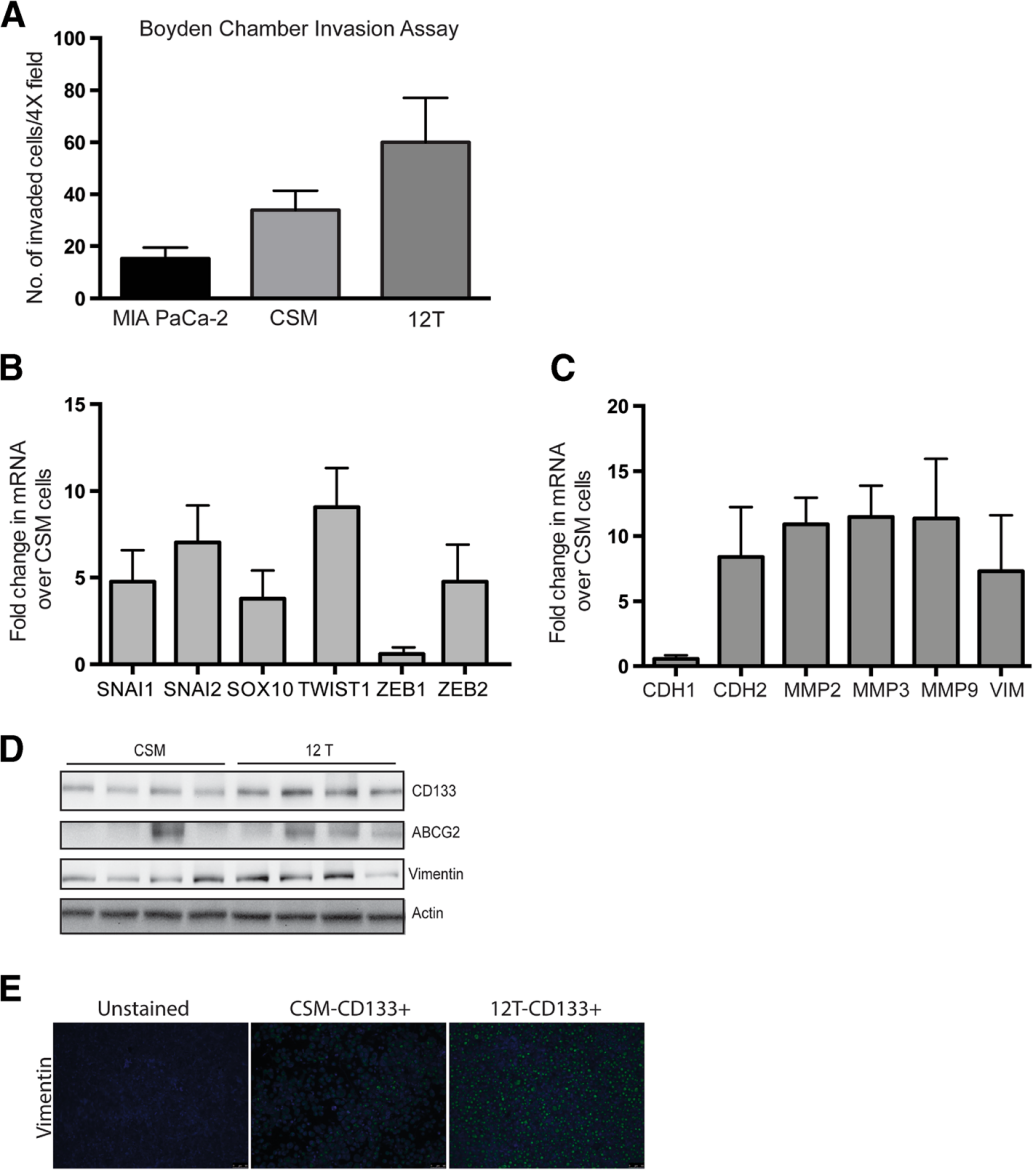
**a** Boyden chamber assay showing increased invasion of 12 T cells compared to CSM cells. RNA Expression of transcription factors involved in **b** EMT and **c** cytoskeletal re-corganization gene expression in 12 T cells compared to CSM cells. **d** Protein expression showing positive correlation of CD133, ABCG2 and Vimentin in CSM CD133^+^ and 12 T CD133^+^ tumor bearing animals and **e** immunofluorescent staining of Vimentin tumor expression. The error bars represent SEM (*n* = 4; * = *p* < 0.05)

Increased CD133 expression has been correlated with increased invasiveness [14, 33]. To test this, we compared CSM cells with 12 T cells for their invasiveness by a Boyden chamber invasion assay (Fig. 4a). 12 T cells were 3 times more invasive compared to the CSM cells. To confirm this further, we looked for expression of EMT genes using a PCR array. Consistent with the invasion assay, the 12 T cells showed increased expression of a number of genes involved in EMT. Transcription factors regulating EMT (SNAI1, SNAI2, SOX10, TWIST1, ZEB1, ZEB2) were overexpressed in 12 T genes when compared with the CSM cells (Fig. 4b). EMT is also associated with a re-organization of cytoskeletal proteins. Classically, expression of CDH1 is downregulated at the onset of EMT and the expression of CDH2 is increased. In keeping with this, we found that CDH1 was downregulated in 12 T cells while CDH2 was upregulated. In agreement with our data showing increased invasiveness of 12 T cells compared to controls, increase in the matrix metalloproteases (MMP2/3/9) involved in extracellular matrix reorganization of the tumor microenvironment during EMT were found to be upregulated in 12 T cells (Fig. 4c). To confirm these further, we looked for expression of classical EMT markers like Vimentin in the tumors developed from 12 T and CSM cells. As seen *in vitro*, the tumor cells developed from 12 T showed increased expression for Vimentin compared to CSM cells (Fig. 4d, e). Vimentin expression also correlated with CD133 and ABCG2 protein expression (Fig. 4d). The densitometric quantitation of the protein bands confirmed this observation (Additional file 4: Figure S1).

### Tumors derived from CD133^+^ sorted cells respond to Minnelide

**Fig. 5:**
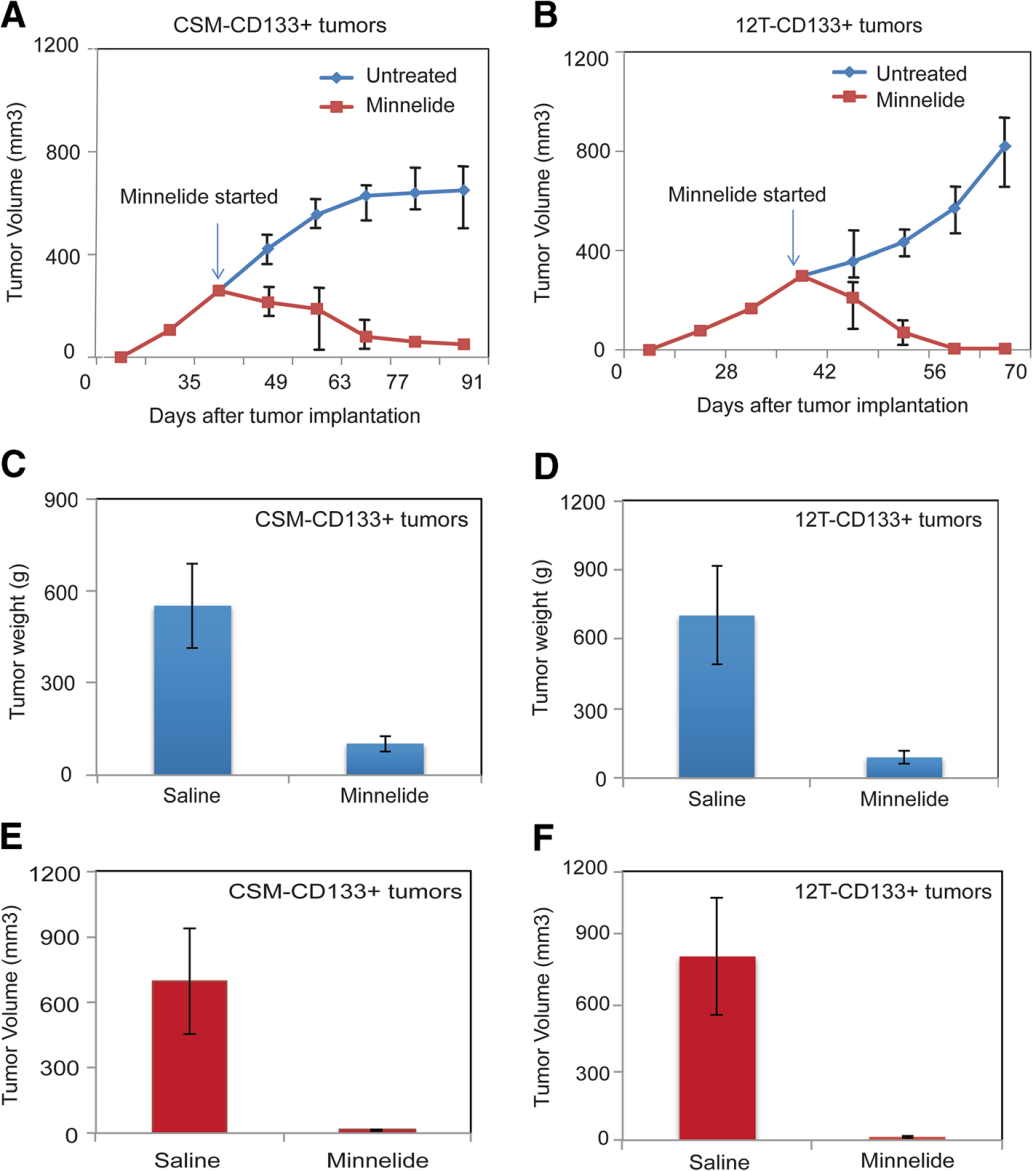
Tumor progression after implantation of **a** CSM-CD133^+^ cells and **b** 12 T-CD133^+^ cells and treatment with Minnelide (0.42 mg/kg body weight) in athymic nude mice. Tumor weight of **c** CSM-CD133^+^ tumors and **d** 12 T-CD133^+^ tumors and tumor volume of **e** CSM-CD133^+^ tumors and **f** 12 T-CD133^+^ tumors after treatment with Minnelide. The error bars represent SEM (*n* = 4; * = *p* < 0.05)

We have previously shown that Minnelide, a prodrug of triptolide, decreases CD133^+^ stem cell population in the KPC mouse model of pancreatic cancer [33]. To further establish if tumors derived from CD133^+^ cells in human cell lines respond to Minnelide, we implanted CD133^+^ cells from CSM and 12 T into the flanks of 20 athymic nude mice (*n* = 10 for each cohort). Once the tumor volume reached 250 mm^3^, mice from each cohort were randomized and one cohort was treated with Minnelide. Following 2 weeks of treatment, tumor regression was observed in both CSM-CD133^+^ group (Fig. 5a) as well as 12 T-CD133^+^ groups, and by the end of the study, tumors from both the group had completely regressed. Tumor weight and tumor volume measurements at the end of the study showed significant reduction in tumor weight (Fig. 5c, d) and tumor volume (Fig. 5e, f).

## Discussion

Accumulating evidence demonstrates that cells from human primary tumors and several cancer cell lines are heterogeneous and hierarchically organized [34, 35]. A small proportion of tumor cells, designated cancer stem cells (CSCs) or tumor-initiating cells, appear to be associated with the expression of distinct cell surface markers, tumorigenicity and resistance to conventional chemotherapy and radiotherapy [4]. CSCs have properties similar to normal stem cells, such as the ability to self-renew, show drug-efflux, slow cell cycling and differentiate into other cell phenotypes. Moreover, CSCs appear to be correlated with malignance, metastasis, poor prognosis and long-term recurrence [36, 37]. The heterogeneity of tumors is further demonstrated by the presence of a subset of tumor cells which is able to rapidly efflux lipophilic fluorescent dyes to produce a characteristic profile based on fluorescence-activated flow-cytometric analysis. These cells are referred to as Side Population (SP) cells. SP cells show enrichment for stem-like properties, are able to initiate tumors, and are also resistant to several anti-tumor compounds [38].

It has been suggested that increased exposure to chemotherapeutic compounds resulted in a drug “tolerant” population of cells that show “stemness” properties [39]. Treatment of pancreatic cancer cell lines with low dose of gemcitabine has shown enrichment of “stem-like” cancer cells [40, 41]. Cancer stem cells or tumor initiating cells have been known to become resistant to therapy with continued exposure to chemotherapeutic compounds owing to increased expression of drug transporters like ABC transporters [41, 42]. Interestingly, overexpression of cancer stem cell marker CD133 has been reported to result in up-regulation of the ABC transporters like ABCG2 [13, 14, 43, 44]. Increased expression of ABCG2 is also characterized by increased drug efflux property of the cell, increased chemo-resistance, and increased invasion [14, 45, 46].

In contrast to many conventional drugs, some treatments have been found to induce cell death in tumor-initiating or side population cells with efficacy in pancreatic cancer [32, 47]. Minnelide, the pro-drug of triptolide, a compound derived from a Chinese herb, has shown immense promise in a number of pre-clinical studies. Minnelide is currently undergoing phase I clinical trials at the University of Minnesota- Minneapolis, MN and Mayo Clinic- Scottsdale, AZ, with promising initial responses.

Our previously published study demonstrated that even after treatment with Minnelide is discontinued, the pancreatic tumors do not grow back [47]. This indicated that treatment with Minnelide might be having a deleterious effect on the tumor initiating cells (TICs) in pancreatic cancer. Our earlier results show that treatment with Minnelide is able to significantly reduce the CD133^+^ population in the spontaneous KPC (Kras^G12D^,Trp53 ^R172H^,Pdx-1-Cre) murine model tumors and in human patient tumor derived xenografts [33]. Further, tumors derived from CD133^+^ population in KPC tumor derived cell line (KPC001) responded to Minnelide as well [33]. These observations were extremely promising. Interestingly, when CD133 was overexpressed in MIA PaCa-2 cells (that have a negligible CD133 expression), we observed increased tumorigenicity in animals [14]. This indicated that CD133 was only just a surface marker that could be used to identify pancreatic TIC, but a surface molecule with complex signaling activity in a cell that resulted in the “stemness” property. When tumors derived from these cells was treated with Minnelide, they responded positively as well (Additional file 5: Figure S2).

In the current study, treatment with a very low dose of triptolide (12.5 nM) resulted in selection of a population of cells that were enriched for surface marker CD133 and expression of the drug transporter ABCG2 (Figs. 1 and 2). Additionally, these cells had increased expression of stemness markers and showed tumorigenic potential at very low concentration (Fig. 2). Increased expression of CD133 is reported to correlate with increased invasiveness [25, 48]. Consistent with this, our studies show that 12 T cells had increased invasiveness compared to CSM cells (Fig. 3). An analysis of the genes involved in Epithelial-Mesenchymal Transition (EMT) revealed overexpression of genes that down-regulate the epithelial phenotype and up-regulate the mesenchymal phenotype in a cancer cell (Fig. 3). Our studies also revealed that expression of CD133, ABCG2 and invasive markers like Vimentin, positively correlated with each other. Since low dose triptolide treatment resulted in enrichment of CD133^+^ population, we further tested this population for its “stem-like” properties. CD133^+^ cells formed colonies *in vitro* and also showed sphere formation *in vitro*. In vivo, the CD133^+^ population from the triptolide treated 12 T cells initiated tumors that faster growth than the CD133^+^ population from CSM cells (Fig. 4).

The current study thus established an “*in vitro* model” for studying the properties associated with cancer stem cells or tumor initiating cells. As seen in cancer stem cells isolated from tumor xenografts, the 12 T cells showed similar enrichment of surface markers CD24/CD44/ESA and CD133. These cells also showed an increased expression of a number of developmental pathways associated with stemness like the hedgehog and the Notch pathways. Many CSCs are characterized by increased expression of EMT genes and drug transporters like ABC transporters. The 12 T cells, which were treated with triptolide for an extended period, showed similar increased expression of EMT along with increased invasiveness. Similarly, these cells also showed an increased expression of ABCG2 and an increased DNA dye efflux (as a measure of increased transporter activity). All these indicated that treatment with a low dose of triptolide *in vitro* resulted in enrichment of a cancer “stem-like” population was also relatively drug resistant, as reflected by their IC50 values (Additional file 6: Figure S3), and when implanted in animals formed tumors and responded to Minnelide (Fig. 5). Our previously published results indicate that though the CD133^+^ cells were generally resistant to both of the standard chemotherapeutic drugs (gemcitabine, 5FU and paclitaxel), they were typically responsive to Minnelide [33].

## Conclusion

Recurrence of pancreatic tumors following surgical resection contributes to the poor survival rate of patients diagnosed with this disease. As tumor recurrence is attributed to the presence of cancer stem-like cells in a tumor, the evaluation of any drug against this population is of utmost importance. This study, in addition to our previous work in a syngeneic murine model, provide compelling evidence for Minnelide treatment against pancreatic cancer initiating cells. Minnelide has shown promise in pre-clinical evaluations and is currently undergoing Phase 1 clinical trials.

## Methods

### Cells and reagents

Pancreatic cancer cells MIA PaCa-2, AsPC-1 was obtained from ATCC. S2-013 was a cell line derived from the metastatic SUIT-2 cell line and was a gift from Professor Masato Yamamoto.

MIA PaCa-2 was grown in DMEM-High Glucose with 10 % FBS, S2-013 was grown and cultured in RPMI with 10 % FBS and AsPC-1 was maintained and cultured in 20 % FBS. All cell growth media included 1X Penicillin-Streptomycin.

EGF, FGF, and B12 supplements were obtained from Life technologies. CD133 magnetic beads were obtained from Miltenyi Biotech, CD24, CD44, ESA were purchased from BD Biosciences. Ki-67 antibody was purchased from Thermo Fisher.

### Generation of triptolide tolerant cells

Pancreatic cancer cells MIA PaCa-2, S2-013 and AsPC-1 were treated with 12.5 nM triptolide (in 10 % FBS-containing medium) for 7 days. Following treatments, the viable, triptolide tolerant cells were allowed to recover in a drug free serum containing media for 48 h. The cells were next transferred to a Cancer Stem Cell medium (CSM):F12:DMEM supplemented with EGF, FGF, B27 Supplement along with fungizone and penicillin-streptomycin. These treated cells were referred to as 12 T. In parallel, another batch of cells was grown in full medium without triptolide and was referred to as CSM. 12 T and CSM cells were used for various cell and animal experiments.

### Flow cytometric analysis of isolated samples

Cells were washed once in PBS and gently scraped into 15 mL centrifuge tubes, washed in PBS prior to staining and stained with the following directly conjugated monoclonal antibodies in the presence of FcR blocking reagent (Miltenyi Biotech): anti-mouse CD133-PE (Miltenyi Biotech), anti-mouse CD44-FITC (BD Biosciences), ESA-APC (BD Bioscience) and CD24-PE (BD Biosciences, USA). IgG isotype controls corresponding to each directly conjugated fluorophore were utilized to identify, quantify, and positively select desired cell populations. All FACS analyses were performed on a BD FACS Canto II (BD Biosciences) using FACS Diva (BD Biosciences) and FlowJo (Tree Star, Ashland, Oregon) software. Debris and cell clusters were excluded during side-scatter and forward-scatter analyses.

### RNA and protein analysis

For protein analysis, the cells were lysed in RIPA buffer (Boston Bioproducts) containing protease inhibitors (Roche) on ice for 15 min. The protein in the cell lysate was quantitated using the BCA protein quantitation kit (Pierce). Equal amount of protein for CSM and 12 T population was then separated on a SDS PAGE and transferred to nitrocellulose membrane. Expression of different proteins following treatment was studied using western blotting and hybridization.

For transcript analysis, RNA was isolated from this population using Trizol (invitrogen) according to manufacturer’s protocol. Real time PCR analysis was performed after synthesizing cDNA (Applied Biosystems) in an ABI7300 instrument (Applied Biosystems).

EMT PCR array (SA Biosciences) was used to study expression level of genes involved in EMT according to manufacturers instruction.

### Animal experiment

All animal experiments were performed according to the University of Minnesota Animal Care Committee guidelines. Sorted cells were washed with serum-free HBSS and suspended in serum free-DMEM/Matrigel mixture (1:1 volume). Either 1000 or 10,000 cells (CSM, 12 T sorted for CD133^+^ or CD133^−^) were injected subcutaneously into the right and left flank of age and gender matched C57BL/6 mice (Jackson Laboratories). 10 mice were used in each group. Tumor were measured weekly and volume was calculated by the formula: 0.52 x l x w^2^. Tumors were allowed to grow until they reached a volume of 1 cm^3^, at which the mice were sacrificed and the tumor tissue was harvested and processed for flow cytometry, immunohistochemistry, protein or RNA experiments.

### Treatment with Minnelide

Minnelide treatment was started 5 days after tumor implantation. 0.42 mg Minnelide/kg body weight was administered intra-peritoneally QD for 25 days. Tumors were measured as stated above. At the end of study, animals were sacrificed according to the University of Minnesota Animal Care guidelines.

### Side population assay/DNA dye efflux assay

Side population analysis was done according to the protocol of Goodell et al [49]. Briefly, cells (1X 10^6^ cells/mL) were incubated in pre-warmed DMEM/5 % FBS containing freshly added Hoechst 33342 (5 μg/ mL final concentration) for 90 min at 37^0^C with intermittent mixing. In some experiments, cells were incubated with the Hoechst dye in the presence of verapamil (50 μmol/L) At the end of incubation, cells were spun down at 4 **°**C and resuspended in ice-cold PBS. PI (2 μg/mL final concentration) was added for 5 min before fluorescence-activated cell sorting (FACS) analysis, which allows for the discrimination of dead versus live cells. The Hoechst dye was excited with the UV laser at 351 to 364 nm and its fluorescence measured with a 515-nm side population filter (Hoechst blue) and a 608 EFLP optical filter (Hoechst red).

### Statistical analysis

Values are expressed as the mean ± SEM. All *in vitro* experiments were performed at least three times. Statistical significance of results was calculated using the Student’s t-test. Columns represent Mean; bars represent Standard Error (*n* = 4; * = *p* < 0.05).

## Additional files

Additional file 1: Table S1. Expression of stemness genes in CSM and 12 T cells (Fold change in expression over CSM cells). SEM is represented within parenthesis. (DOC 30 kb) Additional file 2: Table S2. Tumorigenicity with MIA PaCa-2, CSM and 12 T cells. (DOC 28 kb) Additional file 3: Table S3. Tumorigenicity with CSM-CD133^+^ and 12 T-CD133^+^ cells. (DOC 27 kb) Additional file 4:
**Densitometric quantification of western blot bands of CD133, ABCG2, and Vimentin.** (TIF 973 kb) Additional file 5:
**Response of tumors derived from MIA PaCa-2 cells overexpressing CD133 to Minnelide treatment.** (TIF 788 kb) Additional file 6:
**IC50 values for triptolide of MIA PaCa-2 and CD133** ^**+**^ **and CD133** ^**-**^ **of CSM and 12T groups.** (TIF 705 kb)

## Abbreviations

PDAC: Pancreatic Adenocarcinoma
CSC: Cancer Stem Cells
TIC: Tumor initiating cells
CSM: Cancer Stem cell Medium
FACS: Fluorescence Activated Cell Sorting
