# Supplementary material for: Minnelide effectively eliminates CD133+ side population in pancreatic cancer
Source: Mol Cancer. 2015 Nov 23;14:200. doi: 10.1186/s12943-015-0470-6 (PMC4657383; doi:10.1186/s12943-015-0470-6)
Supplement: Additional file 1: Table S1. — Expression of stemness genes in CSM and 12 T cells (Fold change in expression over CSM cells). SEM is represented within parenthesis. (DOC 30 kb) [file 12943_2015_470_MOESM1_ESM.doc]

Additional file 1: Table S1. Expression of stemness genes in CSM and 12T cells (Fold change in expression over CSM cells). SEM is represented within parenthesis.

|  | MIA PaCa-2 | S2-013 | AsPC-1 |
| --- | --- | --- | --- |
| Sox2 | 2.5 (+/-0.03) | 3.4 (+/-0.02) | 3.8 (+/-0.05) |
| Nanog | 3.1(+/- 0.02) | 4.2(+/-0.1) | 2.9(+/- 0.03) |
| Oct4 | 2.2(+/- 0.1) | 2.3(+/-0.02) | 3.1(+/- 0.07) |
| Notch1 | 5.5 (+/-0.1) | 4.3(+/-0.02) | 4.4(+/-0.05) |
| Jagged | 4.8 (+/- 0.02) | 5.1(+/- 0.08) | 4.8(+/-0.02) |
| Ptch | 6.2 (+/- 0.05) | 5.4(+/- 0.1) | 6.1(+/-0.1) |
| Gli | 3.1 (+/- 0.03) | 2.8 (+/- 0.02) | 2.2(+/-0.05) |
| Shh | 2.5 (+/- 0.02) | 3.1 (+/-0.03) | 2.3 (+/-0.02) |
