# Supplementary material for: Minnelide effectively eliminates CD133+ side population in pancreatic cancer
Source: Mol Cancer. 2015 Nov 23;14:200. doi: 10.1186/s12943-015-0470-6 (PMC4657383; doi:10.1186/s12943-015-0470-6)
Supplement: Additional file 2: Table S2. — Tumorigenicity with MIA PaCa-2, CSM and 12 T cells. (DOC 28 kb) [file 12943_2015_470_MOESM2_ESM.doc]

Additional file 2: Table S2. Tumorigenicity with MIA PaCa-2, CSM and 12T cells.

|  | Number of animals in study | Tumor take at the end of study  (No. of tumor bearing mice/total no. of mice) |
| --- | --- | --- |
| MIA PaCa-2 | 5 | 0/5 |
| CSM-1000 | 9 | 1/9 |
| CSM-10000 | 9 | 2/9 |
| 12T-1000 | 9 | 4/9 |
| 12T-10000 | 8 | 7/8 |
