# Supplementary material for: Minnelide effectively eliminates CD133+ side population in pancreatic cancer
Source: Mol Cancer. 2015 Nov 23;14:200. doi: 10.1186/s12943-015-0470-6 (PMC4657383; doi:10.1186/s12943-015-0470-6)
Supplement: Additional file 3: Table S3. — Tumorigenicity with CSM-CD133+ and 12 T-CD133+ cells. (DOC 27 kb) [file 12943_2015_470_MOESM3_ESM.doc]

Additional file 3: Table S3. Tumorigenicity with CSM-CD133^+^ and 12T-CD133^+^ cells.

|  | Number of animals in study | Tumor take at the end of study  (No. of tumor bearing mice/total no. of mice) |
| --- | --- | --- |
| CSM-CD133^-^ | 10 | 0/10 |
| CSM-CD133^+^ | 10 | 7/10 |
| 12T-CD133^-^ | 10 | 0/10 |
| 12T-CD133^+^ | 10 | 10/10 |
