# Supplementary figures and images for: Minnelide effectively eliminates CD133+ side population in pancreatic cancer
Source: Mol Cancer. 2015 Nov 23;14:200. doi: 10.1186/s12943-015-0470-6 (PMC4657383; doi:10.1186/s12943-015-0470-6)

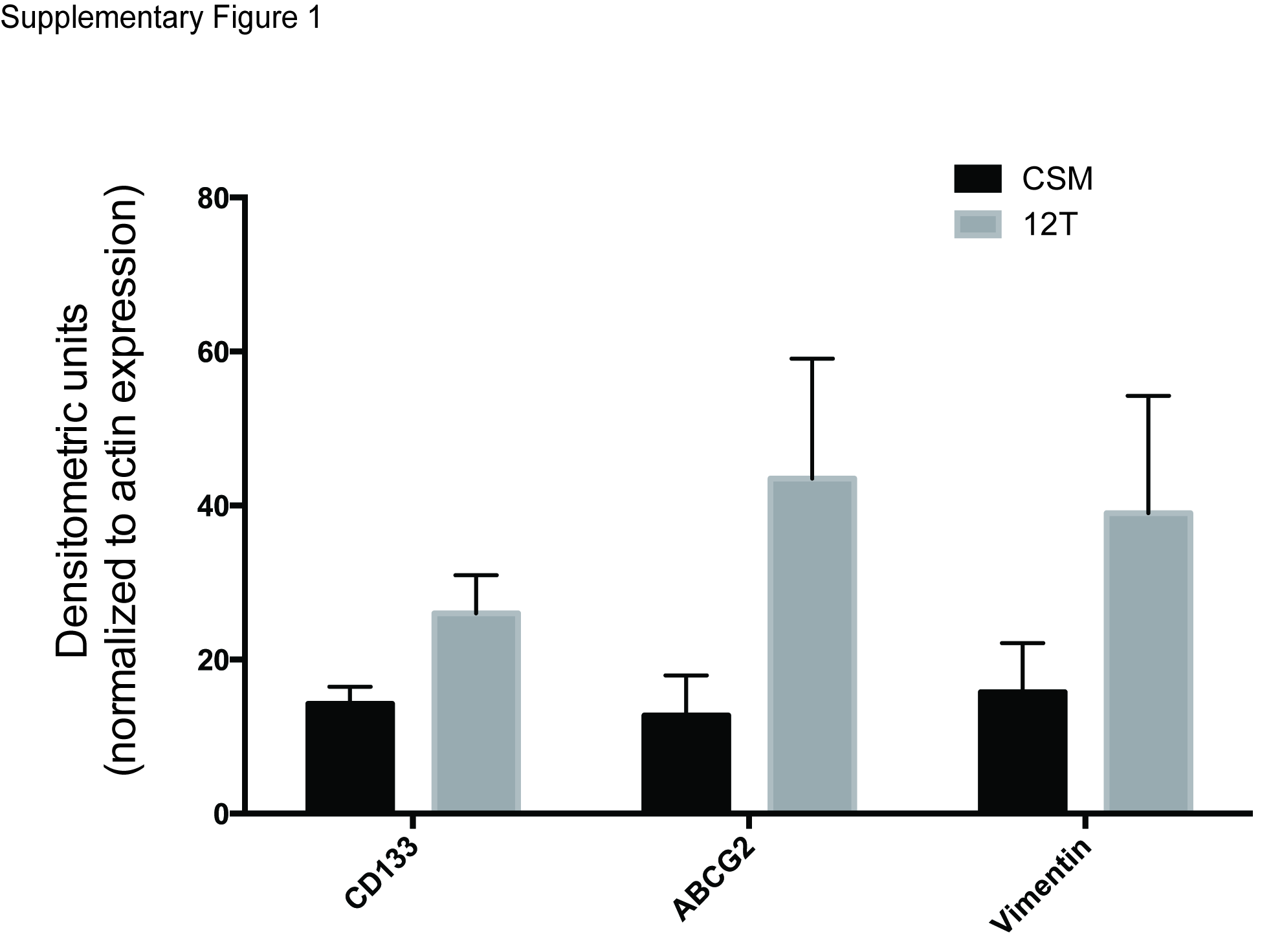

Supplement: Additional file 4: — Densitometric quantification of western blot bands of CD133, ABCG2, and Vimentin. (TIF 973 kb) [file 12943_2015_470_MOESM4_ESM.tif]

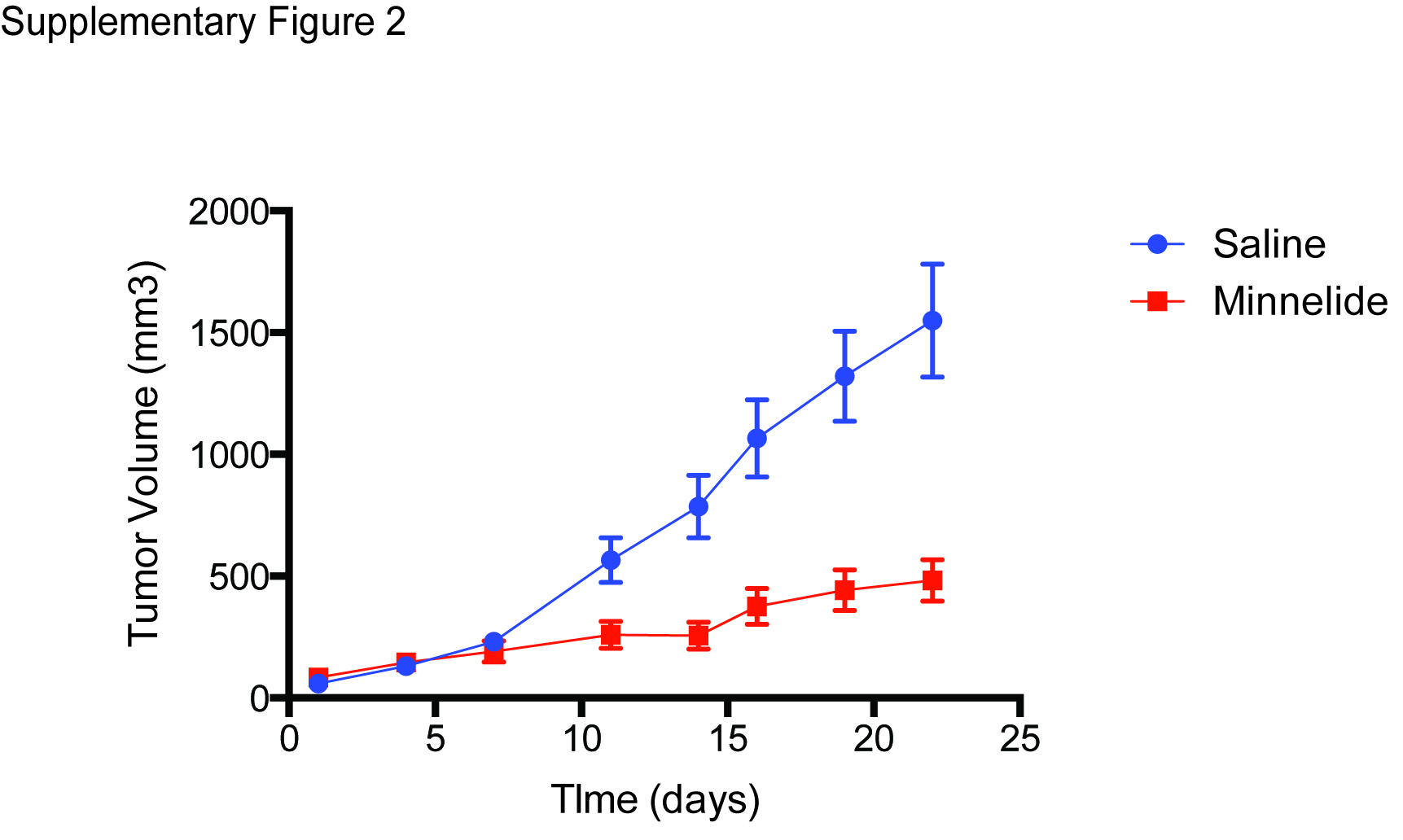

Supplement: Additional file 5: — Response of tumors derived from MIA PaCa-2 cells overexpressing CD133 to Minnelide treatment. (TIF 788 kb) [file 12943_2015_470_MOESM5_ESM.tif]

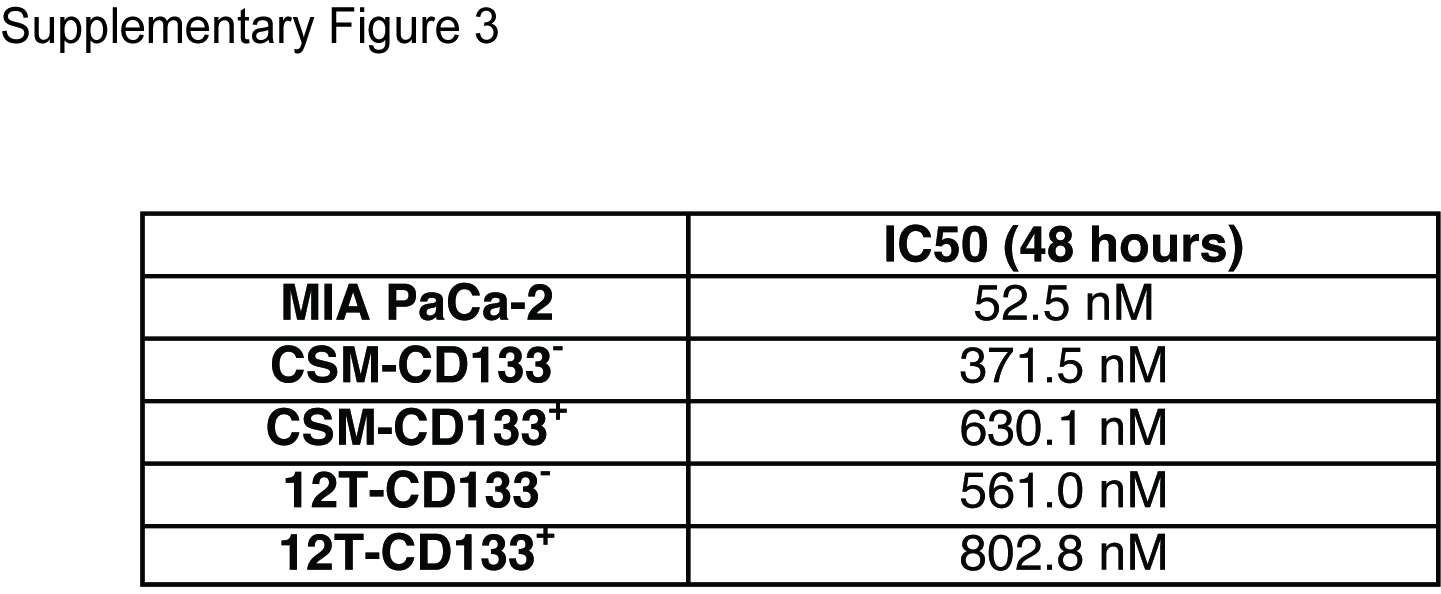

Supplement: Additional file 6: — IC50 values for triptolide of MIA PaCa-2 and CD133 + and CD133 - of CSM and 12T groups. (TIF 705 kb) [file 12943_2015_470_MOESM6_ESM.tif]
